# Involvement of Intracellular and Mitochondrial Aβ in the Ameliorative Effects of Huperzine A against Oligomeric Aβ_42_-Induced Injury in Primary Rat Neurons

**DOI:** 10.1371/journal.pone.0128366

**Published:** 2015-05-29

**Authors:** Yun Lei, Ling Yang, Chun Yan Ye, Ming Yan Qin, Huai Yu Yang, Hua Liang Jiang, Xi Can Tang, Hai Yan Zhang

**Affiliations:** 1 CAS Key Laboratory of Receptor Research, Shanghai Institute of Materia Medica, Chinese Academy of Science, Shanghai, China; 2 Drug Discovery and Design Center, State Key Laboratory of Drug Research, Shanghai Institute of Materia Medica, Chinese Academy of Sciences, Shanghai, China; North Carolina A&T State University, UNITED STATES

## Abstract

Considerable studies indicate huperzine A is a promising natural product to suppress neuronal damages induced by β-amyloid (Aβ), a key pathogenic event in the Alzheimer’s disease (AD). As an extension, the present study for the first time explored whether the beneficial profiles of huperzine A against oligomeric Aβ_42_ induced neurotoxicity are associated with the accumulation and detrimental function of intraneuronal/mitochondrial Aβ, on the basis of the emerging evidence that intracellular Aβ is more relevant to AD progression as compared with extracellular Aβ. Huperzine A treatment was shown to significantly attenuate the neurotoxicity of oligomeric Aβ_42_, as demonstrated by increased neuronal viability. Interestingly, our results proved that exogenous Aβ_42_ could accumulate intraneuronally in a dose- and time-dependent manner, while huperzine A treatment markedly reduced the level of intracellular Aβ_42_. Moreover, huperzine A treatment rescued mitochondrial dysfunction induced by oligomeric Aβ_42_, including adenosine triphosphate (ATP) reduction, reactive oxygen species (ROS) overproduction and membrane potential depolarization. Further study demonstrated that huperzine A also significantly reduced the level of Aβ_42_ in the mitochondria-enriched subcellular fractions, as well as the Aβ_42_ fluorescent signals colocalized with mitochondrial marker. This study indicates that interfering intracellular Aβ especially mitochondrial Aβ accumulation, together with ameliorating Aβ-associated mitochondrial dysfunction, may contribute to the protective effects of huperzine A against Aβ neurotoxicity. Above results may shed more light on the pharmacological mechanisms of huperzine A and provide important clues for discovering novel therapeutic strategies for AD.

## Introduction

AD, the most common cause of dementia, is a chronic disorder characterized by progressive decline in cognitive function [1]. Compelling evidence shows that excessive accumulation of Aβ peptide in the brain is a central and perhaps defining event in the pathogenesis of AD [2, 3], thus “Aβ cascade hypothesis” is strongly supported by evidence of Aβ-related pathology and has been the focal point of research and pharmaceutical industry for the past twenty years [2, 4]. Although the precise molecular mechanisms behind Aβ-associated neurotoxicity remain to be elucidated, increasing evidence indicates that intracellular accumulation of Aβ is more relevant to AD process as compared with extracellular Aβ [5, 6]. Intracellular Aβ has been proposed as an early event in AD pathogenesis, which is built up by intracellular Aβ generation and reuptake of secreted Aβ from extracellular environment [7]. Recent researches show that intracellular Aβ plays a pivotal role in AD-associated malignant changes, including tau phosphorylation [8], reduction in synaptic protein expression [9], and mitochondrial dysfunction [10]. Afore-mentioned evidence has raised the prospect of discovering effective therapeutic strategies on blocking Aβ internalization from the extracellular space or its intracellular accumulation, especially under the circumstance that current pharmacological approaches against Aβ formation or immunization failed to gain promising efficacy on preventing and/or delaying AD progressing.

Huperzine A, a *Lycopodium* alkaloid isolated from Chinese folk medicine *huperzia serrata* (Qian Ceng Ta), has been proven to effectively attenuate cognitive deficits and is widely used for AD treatment in China [11, 12]. Recent studies indicate that huperzine A can effectively alleviate Aβ-associated neurotoxicity in many *in vitro* and *in vivo* models, besides its potent acetylcholinesterase (AChE) inhibitory effect [12, 13]. Huperzine A has been proven to rescue Aβ_25–35_ induced oxidative stress and apoptosis in rat pheochromocytoma cells [14], NG108-15 cells [15], and rat cortical neurons [16]. Nevertheless, the precise molecular mechanisms underlying the protective effects of huperzine A against Aβ-associated neuronal dysfunction have not been well clarified yet. As strong evidence accumulated in recent years indicates that Aβ oligomers, rather than Aβ fibrils or monomers, are the prominent neurotoxins in AD pathology [17–19], the current work for the first time employed oligomeric Aβ_42_ to determine the ameliorative effect of huperzine A against Aβ-induced injury in primary cortical neurons and explore whether this effect is associated with intracellular Aβ.

Recently, a number of studies have brought up the close association between intracellular Aβ and mitochondrial dysfunction [20]. Intracellular Aβ can be taken up by mitochondria through the TOM machinery and MAM connection [21], and hence mitochondria become one of the major subcellular pools of Aβ [22]. Moreover, many studies have provided substantial evidence for the direct links between progressive accumulation of mitochondrial Aβ and mitochondrial dysfunction [20, 23], including abnormal ATP production, oxidative stress and membrane potential damage [24]. Taken together the potential effects of huperzine A on mitochondrial dysfunction in various models [14, 16], it will be therefore of great interest to clarify whether the mitochondrial function and mitochondrial Aβ level are also influenced by huperzine A treatment in oligomeric Aβ_42_-associated primary cortical neuronal model.

## Materials and Methods

### Ethics Statement

The animal works and experiment protocols were approved by the Institutional Animal Care and Use Committee of Shanghai Institute of Materia Medica. All the Sprague Dawley (SD) rats were obtained from Shanghai Laboratory Animal Center, Chinese Academy of Science, and maintained in the specific pathogen-free and Association for Assessment and Accreditation of Laboratory Animal Care International (AAALAC) approved animal facility under 20–26°C temperature and 40–70% humidity with 12 h light/dark cycles.

### Materials

Huperzine A (purity > 99%, Wan Bang Pharmaceutical Co.Ltd.) was dissolved in 0.1 N HCl at 5 mg/ml as stock solution, and diluted to proper concentrations before usage with neurobasal medium. Stock solution of Aβ_42_ (Millipore) was prepared in 100% DMSO at a concentration of 5 mM, and then diluted to 10 μM with neurobasal medium and aged for 24 hours at 4°C to prepare oligmeric Aβ_42_. The aggregation state of prepared oligmeric Aβ_42_ was characterized by atomic force microscope (AFM) and western blotting (Figure A in S1 File), which showed the similar oligomeric forms as the observation of other labs [25, 26]. All stock solutions were stored at -20°C. All the other chemicals and reagents were purchased from Sinopharm Chemical Reagent Co.Ltd., unless otherwise stated.

### Primary cortical neuron culture and drug treatment

Cortical neurons were isolated from E16-E17 SD rat embryos according to the method described by Lu’s lab [27] with slight modification. Briefly, cortices were dissected in cold high glucose Dulbecco's Modified Eagle's Medium (HG-DMEM, Invitrogen), dissociated with 0.125% (w/v) trypsin at 37°C for 15 minutes, and then triturated with pipette in HG-DMEM with 10% (v/v) fetal bovine serum (FBS, Gibco). After non-dispersed tissue settled for 2 minutes, the supernatants were filtered through strainer (300/400 mesh) and transferred to a new tube. Then, the cell suspension was diluted into optimal concentration (6×10^5^ cells/well for 6-wells plate, 3×10^4^ cells/well for 96-wells plate, 1.5×10^5^ cells/slide) with HG-DMEM containing 10% FBS and plated onto poly-L-lysine (Sangon Biotech)-coated plates or slides. Four hours later, the culture medium was completely removed, and the cells were cultured in neurobasal medium (Invitrogen) with 0.5 mM L-glutamine, 2% (v/v) B27 supplement, penicillin (60 mg/L) and streptomycin (50 mg/L). No mitosis inhibitors were used. Half of the culture medium was refreshed every 3 days. Following 9 days of culture, the purity of primary cultured neurons was assessed by immunocytochemical technique using Tau as neuronal marker and GFAP as glial marker [28], which revealed that the majority of cultured cells were neurons (data not shown). Neurons were pretreated with 1 μM oligomeric Aβ_42_ or HiLyte Fluor 488-labeled-Aβ_42_ (HiLyte-Aβ_42_, AnaSpec) for 2 hours and then incubated with 0, 0.1, 1 or 10 μM huperzine A for another 22 hours, while the vehicle-treated control group was treated with same amount of neurobasal medium.

### MTT assay

After treatment, 3-(4,5-Dimethylthiazol-2-yl)-2,5-diphenyltetrazolium bromide (MTT, Amresco) was added to culture medium at final concentration of 0.5 mg/ml and incubated at 37°C for 3 hours. The medium was removed after incubation, 100 μL/well DMSO was then added to dissolve the formazan. Cell plates were shaken for 5 minutes and the absorbance of each well was measured with a PerkinElmer microplate reader at 490 nm. The absorbance value was normalized against the value of vehicle-treated control group.

### SRB assay

Sulforhodamine B (SRB) assay relies on the ability of SRB binding to protein components. After treatment, cells were fixed with 10% trichloroacetic acid for 1 hour at 4°C. Then, plates were washed with H_2_O and air-dried. Cells were stained with 0.4% SRB in 1% acetic acid for 30 minutes at room temperature. Plates were washed with 1% acetic acid and air-dried. The stained cells were lysed in 10 mM Tris buffer and the absorbance was measured at 550 nm with a PerkinElmer microplate reader. The absorbance value was normalized against the value of vehicle-treated control group.

### Detection of neuronal ATP production

The ATP level of neurons was measured using a bioluminescent ATP detection kit (Promega). Briefly, cells were placed at room temperature for 30 minutes, and then lysed by adding 100 μl of ATP-releasing reagent. The lysates were incubated with the luciferin substrate and luciferase enzyme in the dark for 10 minutes to stabilize the luminescent signal. The intensity of bioluminescence was measured using a PerkinElmer microplate reader. The bioluminescence intensity was normalized by control group and presented as the relative ATP level [23].

### Measurement of ROS level

ROS were measured based on the oxidation of 2', 7'-dichlorodihydrofluorescein diacetate (H_2_DCF-diacetate, Molecular Probes) to fluorescent DCF [29]. After treatment, cells were incubated in darkness with 10 μM H_2_DCF-diacetate for 45 minutes at 37°C in NaCl-medium (132.0 mM NaCl, 4.0 mM KCl, 1.0 mM CaCl_2_, 1.4 mM MgCl_2_, 1.2 mM NaH_2_PO_4_, 6.0 mM glucose, 10.0 mM HEPES, pH 7.4). After two washes with above medium, cultured cells were solubilized with 1% SDS buffer (1% SDS, 5 mM Tris-HCl, pH 7.4). The intensity of DCF fluorescence in the lysate was measured using a PerkinElmer microplate reader with 485 nm excitation and 520 nm emission filters. The fluorescence intensity was normalized by control group and presented as the relative ROS level [30].

### Fluorimetric analysis of mitochondrial membrane potential

Mitochondrial membrane potential was measured using the fluorescent cationic dye JC-1 (Molecular Probes). After treatment, cells were incubated with the JC-1 (0.1 μg/ml) at 37°C for 15 minutes and washed with PBS. The fluorescence intensity of JC-1 aggregates was detected with 520 nm excitation and 590 nm emission filters (red fluorescence), whereas the fluorescence intensity of the JC-1 monomers was measured with 485 nm excitation and 520 nm emission filters (green fluorescence) using a PerkinElmer microplate reader. The fluorescence intensity ratio of aggregates to monomers (red fluorescence to green fluorescence) was calculated as an indicator of mitochondrial membrane potential (ΔΨ_m_). The ΔΨ_m_ was presented as the percentage of control group [31].

### Protein extraction and western blotting

For total cellular protein extraction, cells were washed twice with ice-cold PBS and lysed in RIPA buffer (50 mM Tris-HCl, 150 mM NaCl, 0.5% sodium deoxycholate, 1% Triton X-100, 0.1% sodium dodecyl sulfate, 1 mM NaF, 1 mM Na_3_VO_4_, 1 mM PMSF, 1% P8340, pH 7.4), followed by centrifugation at 12,000 *g* for 15 minutes at 4°C. Protein concentration of supernatants was measured using a BCA assay kit (Pierce).

The protein samples were mixed with Loading buffer without DTT and separated in 4%/10%/17% gradient Tricine-SDS-PAGE gels for Aβ blotting. The proteins were subsequently transferred to the nitrocellulose membrane. Blots were blocked with 5% nonfat milk for 1 hour at room temperature before incubation overnight with primary antibodies: Aβ (6E10, Covance, SIG-39320, mouse monoclonal antibody, 1:1000 dilution), β-actin (Sigma Aldrich, A5441, mouse monoclonal antibody, 1:10000 dilution). Blots were washed with Tris-buffered saline buffer containing 0.05% Tween-20, incubated with a horseradish peroxidase-conjugated anti-mouse antibody (Kangcheng, 1:5000) for 1 hour at room temperature, and then detected using the ECL plus detection kit (Amersham GE Healthcare). The immunoreactive bands were visualized by autoradiography, and the intensity of each band was quantified with Image J software.

### Intracellular and mitochondrial Aβ_42_ level measurement

Intracellular Aβ_42_ level was determined by ELISA kit (Invitrogen) according to the manufacturer’s instruction. The whole cell lysates were diluted 300 times (v/v) with ELISA dilution buffer and used as samples for ELISA. According to our experimental result, the residual amount of RIPA buffer in ELISA samples did not influence the ELISA assay (data not shown).

The mitochondria-enriched fractions were prepared according to the method described by Yan’s lab [32] with slight modification. Briefly, after treatment, cells were washed twice with ice-cold PBS and harvested in ice-cold PBS using flat-cell scrapers, and centrifuged for 5 minutes at 300 *g* (4°C) to collect cells. The cell deposits were gently homogenized 10 times using a Dounce homogenizer in 200 μL sucrose-based lysis buffer (50 mM Tris, 1 mM EDTA, 1 mM EGTA, 250 mM sucrose, 1 mM PMSF, 1% P8340, pH 7.4). The lysates were centrifuged at 750 *g* for 10 minutes (4°C) to remove nuclei and cell debris. The supernatants were centrifuged at 10,000 *g* for another 15 minutes (4°C). RIPA buffer (30 μL) was added to suspend the resulting pellets and the lysate was used as mitochondria-enriched fraction. Centrifuged the resulting supernatants at 105,000 *g* for 60 minutes (4°C). The final supernatants were used as cytosolic fraction. The final pellets were re-suspended with 50 μL RIPA buffer and used as membrane-enriched fraction. The purity of subcellular fractions was detected using western blotting (Figure B in S1 File). After appropriate dilution, the samples and standards were measured according the manual of ELISA kit. Absorbance at 450 nm was measured using a PerkinElmer microplate reader. The protein content of each sample was detected using a BCA assay kit. Aβ_42_ level was calculated from the standard curve and presented as ng/mg protein or the percentage of Aβ_42_-treated group.

### Confocal microscopy observation of intracellular Aβ distribution

After treatment with HiLyte-Aβ_42_, the media were removed and cells were incubated with pre-warmed (37°C) solution containing mitotracker probe (250 nM, Invitrogen) for 45 minutes under growth condition. After washing twice with PBS, the cells were fixed with PBS containing 4% paraformaldehyde. The fixed cells were further incubated with 4', 6-diamidino-2-phenylindole (DAPI, 1 μg/ml) to label nuclei and rinsed three times with PBS. The confocal images were obtained with Olympus FV1000-IX81 microscope, using the vendor-provided software (OLYMPUS FLUOVIEW Ver.2.1b Viewer). Fluorescence intensity was quantified with Image J software and presented as the percentage of HiLyte-Aβ_42_-treated group.

### Statistical analysis

All data were presented as mean ± SEM. Results of MTT assay and SRB assay were analyzed using one-way ANOVA with the Dunnett’s test, and the other results were analyzed using Student’s t-test. For all statistical tests, p < 0.05 was considered significant.

## Results

### Huperzine A alleviated oligomeric Aβ_42_ induced damage in the primary rat neurons

**Fig 1:**
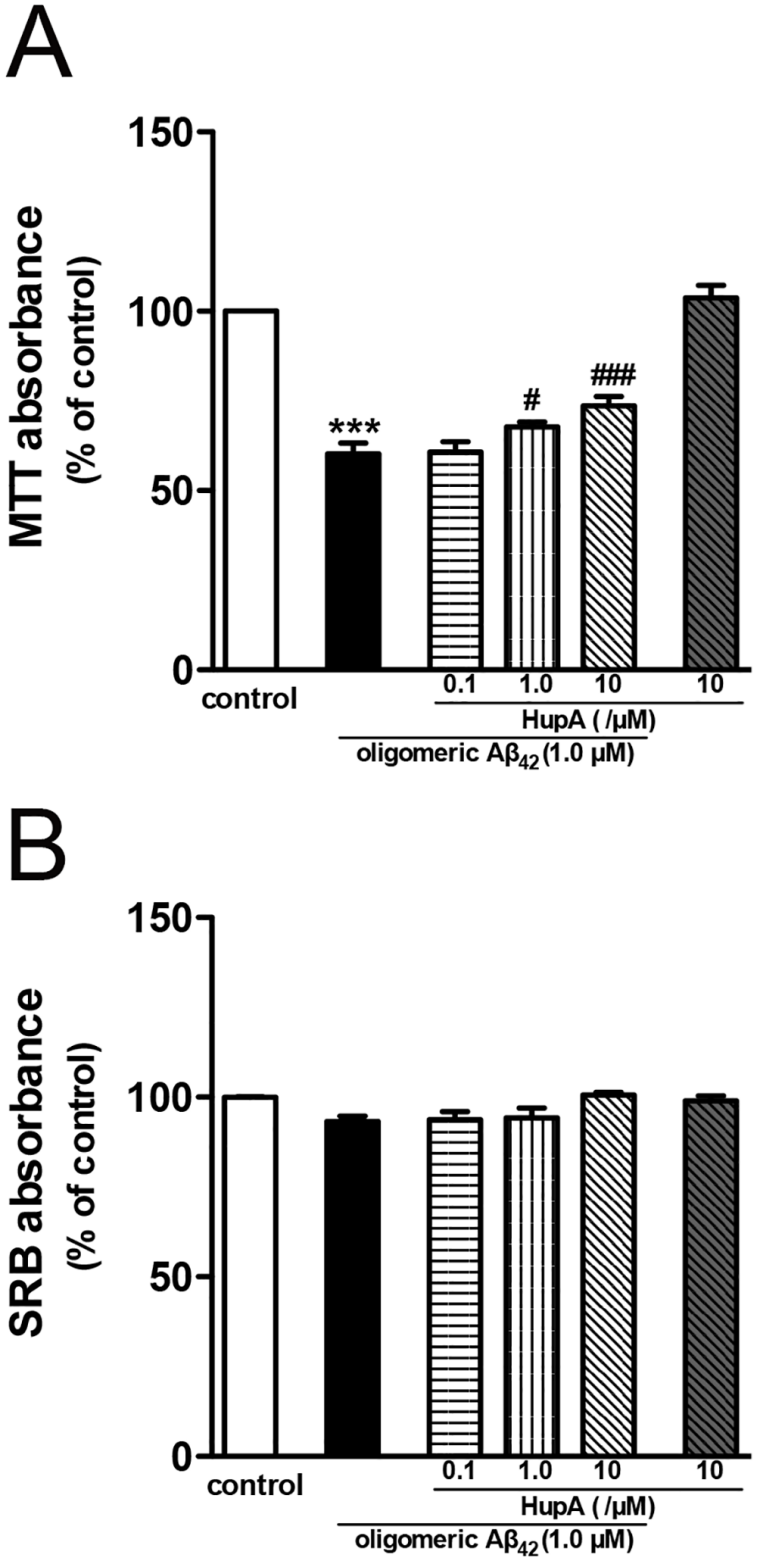
Huperzine A ameliorated oligomeric Aβ_42_ induced damage in primary rat neurons. **A.** The results of MTT assay (N = 6). **B.** The results of SRB assay (N = 3). Data were presented as percentage of control group, and shown as mean ± SEM. ***p < 0.001 vs control group; ^#^p < 0.05, ^###^p < 0.001 vs oligomeric Aβ_42_-treated group. Huperzine A was abbreviated as HupA.

In order to evaluate the effect of huperzine A on oligomeric Aβ_42_ induced neurotoxicity, here we employed MTT assay and SRB assay on primary cortical neurons. The MTT absorbance of neurons decreased to 60% of control group upon incubation with 1 μM oligomeric Aβ_42_ (Fig 1A, p < 0.001 vs vehicle-treated control group), while post-treatment with 1 μM or 10 μM huperzine A markedly increased the MTT absorbance (Fig 1A, p < 0.05 and p < 0.001 vs Aβ_42_-treated group, respectively). No significant difference was observed between control group and huperzine A without oligomeric Aβ_42_-treated group (p > 0.05). As shown in Fig 1B, there was no significant difference of the SRB absorbance between oligomeric Aβ_42_-treated group and vehicle-treated control group (p > 0.05). Huperzine A treatment did not change the SRB absorbance either (p > 0.05). We chose 10 μM huperzine A to carry out the subsequent experiments.

### Huperzine A decreased intracellular Aβ_42_ accumulation

**Fig 2:**
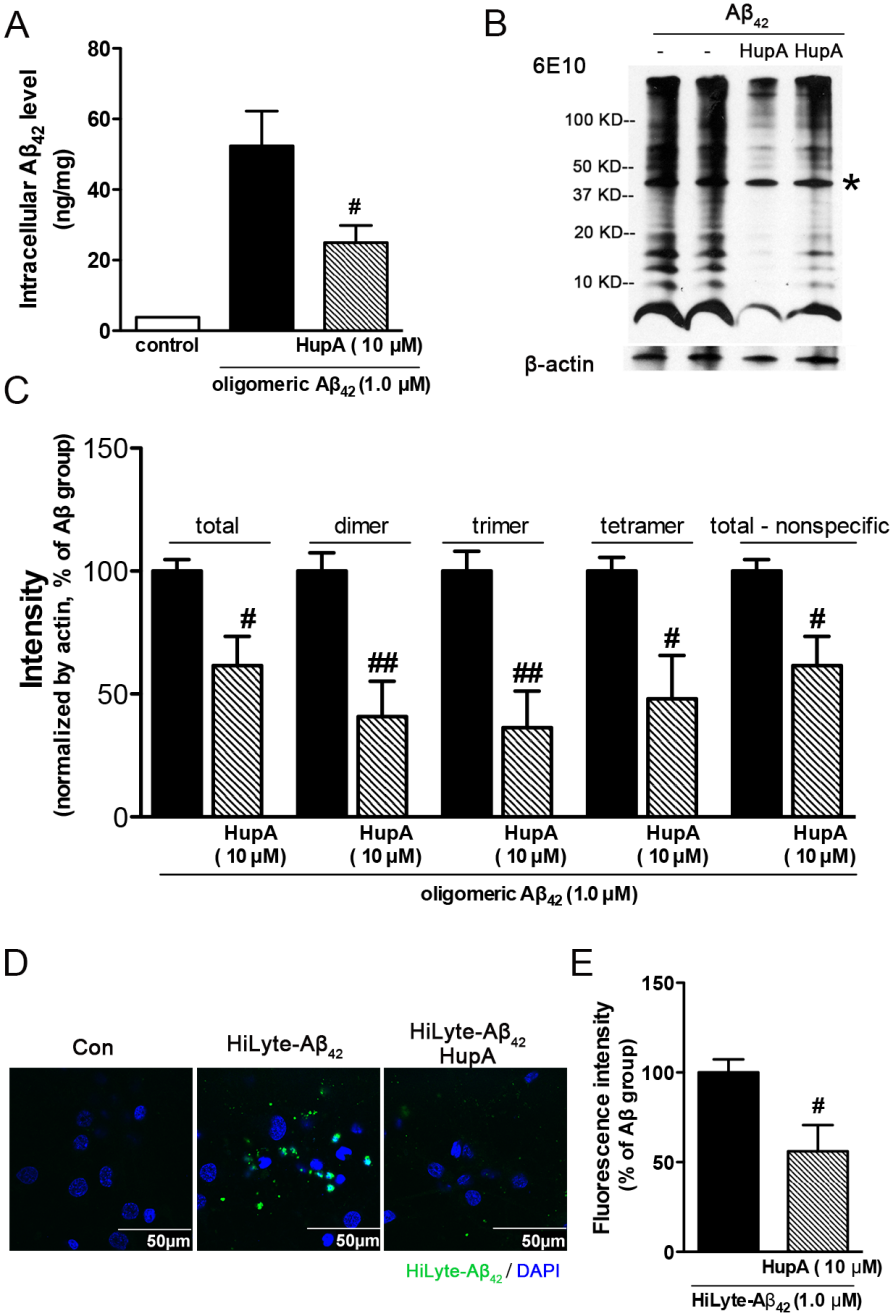
Huperzine A decreased intracellular Aβ_42_ accumulation. Intracellular Aβ_42_ level and/or form were measured by ELISA (A, N = 4) and western blotting (B). C. The statistical result of western blotting (N = 3). D. The representative figures of primary neurons exposed to HiLyte-Aβ_42_. E. The statistical result of HiLyte-Aβ_42_ fluorescence intensity (N = 4). Data were shown as mean ± SEM. Asterisk denoted the non-specific band detected by the 6E10 antibody. ^#^p < 0.05 vs oligomeric Aβ_42_-treated group or HiLyte-Aβ_42_ treated group. Huperzine A was abbreviated as HupA. Scale bar: 50 μm.

Intracellular Aβ plays an important role in Aβ induced neuronal toxicity [7]. To evaluate whether huperzine A could influence the level of intracellularly accumulated Aβ, we measured the level of Aβ in cell lysate by ELISA using an antibody specific for the NH_2_-terminus region of human Aβ (Invitrogen) to distinguish it from endogenous rat Aβ. As shown in Figure C in S1 File, Aβ_42_ accumulated in neurons in a dose- and time-dependent manner, whereas the Aβ_42_ of vehicle-treated neurons was at background level. The level of intracellular Aβ_42_ correlated well with the cell viability (Figure C in S1 File, R^2^ = 0.98, p < 0.001). Huperzine A treatment significantly decreased the level of intracellular Aβ_42_ (Fig 2A, p < 0.05 vs oligomeric Aβ_42_-treated group). We also employed western blotting to evaluate the level and form of Aβ in cell lysate. As shown in Fig 2B and 2C, huperzine A-treated neurons displayed lower Aβ_42_ immuno-intensity as compared with vehicle-treated neurons (p < 0.05 vs oligomeric Aβ_42_-treated group), which was comparable to the results of ELISA. Similar decreasing tendencies were found in the changes of Aβ dimer, trimer and tetramer level (p < 0.01 for dimer and trimer, p < 0.05 for tetramer, Fig 2C), which are the normally recognized toxic forms of synthetic Aβ oligomers [17, 33–35]. Interestingly, there was an obvious band (between 37 kD and 50 kD) existed in the immunoblots of both Aβ exposed alone group and Aβ-exposed with huperzine A post-treatment group, which was marked with asterisk (Fig 2B). Considering this band also existed in the non-Aβ-treated cell lysate (Figure D in S1 File), it is mostly likely to be a nonspecific signal of 6E10 antibody. To be in agreement, similar phenomenon was also observed in other literature [36]. Nevertheless, the existing of this nonspecific band did not affect the experimental outcome as the intensity of total detected bands, including or excluding the nonspecific bands, showed comparable decrease in huperzine A-treated group (Fig 2C). Above results obtained through ELISA and western blotting suggested that huperzine A decreased intracellular Aβ accumulation.

To make further confirmation, we applied HiLyte-Aβ_42_ [37, 38] on neurons. HiLyte-Aβ_42_ was proved to possess similar neuronal toxicity as compared with previous Aβ_42_ in MTT assay (Data not shown). As shown in Fig 2D and 2E, huperzine A treatment decreased the intracellular HiLyte-Aβ_42_ fluorescence intensity (p < 0.05 vs oligomeric Aβ_42_ treated group), which was similar as the results obtained by ELISA and western blotting.

### Huperzine A ameliorated oligomeric Aβ_42_ induced mitochondrial dysfunctions in primary cortical neurons

**Fig 3:**
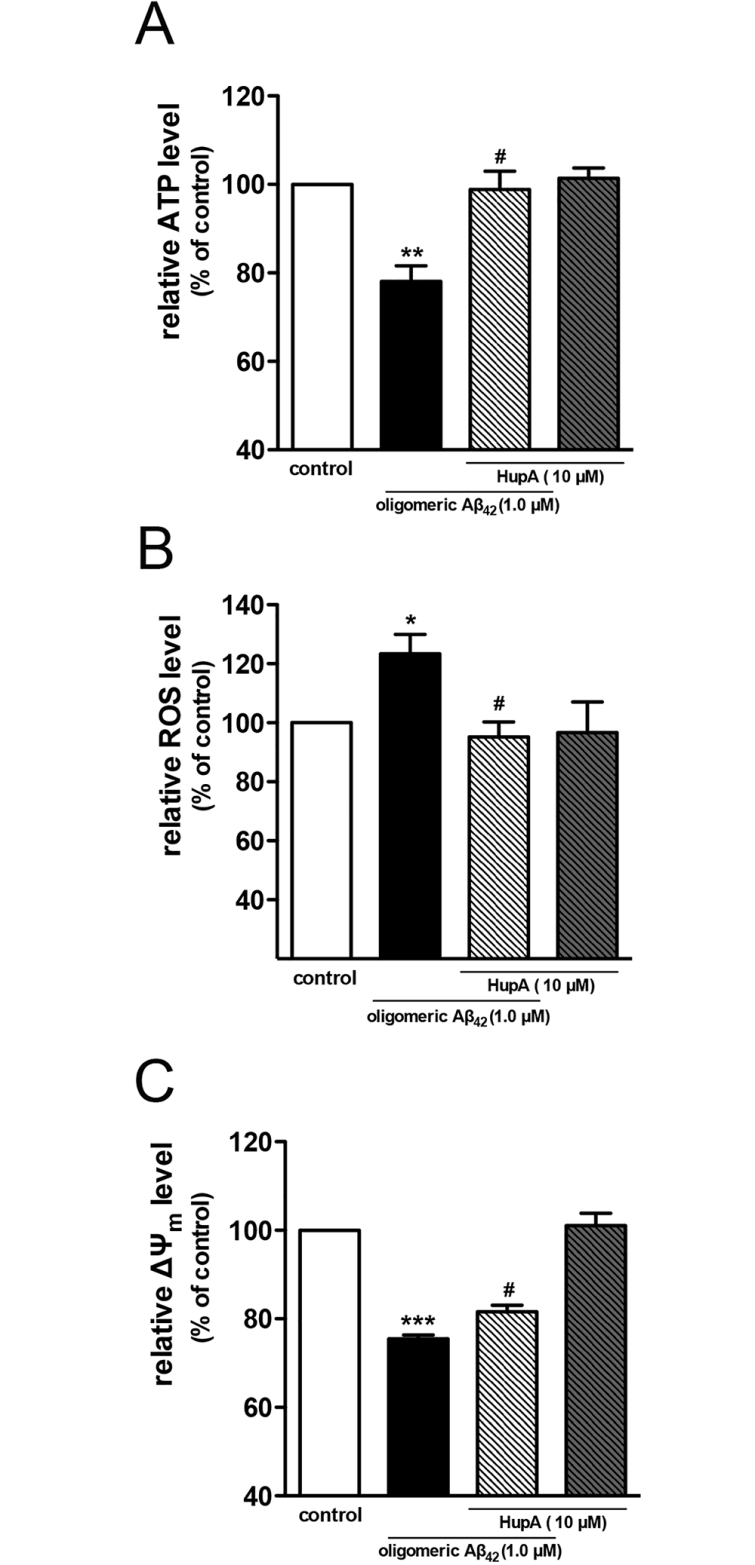
Huperzine A ameliorated oligomeric Aβ_42_ induced ATP reduction (A), ROS overproduction (B) and mitochondrial membrane potential decrease (C). N = 3 for ATP assay, N = 4 for ROS assay, N = 4 for ΔΨ_m_ assay. Data were shown as mean ± SEM. *p < 0.05, **p < 0.01, ***p < 0.001 vs control group; ^#^p < 0.05 vs oligomeric Aβ_42_-treated group. Huperzine A was abbreviated as HupA.

Mitochondrial dysfunction is largely involved in the intracellular Aβ toxicity [22]. We therefore conducted a series of assays to evaluate the mitochondrial function of oligomeric Aβ_42_-exposed neurons treated with or without huperzine A. The cellular ATP level was firstly measured to evaluate the mitochondrial function. As an energy storage and transfer molecule, ATP was mostly synthesized in the mitochondria. Using a luciferin-luciferase assay, we observed that the ATP level of oligomeric Aβ_42_ treated group reduced obviously as compared with the control group (Fig 3A, p < 0.01 vs vehicle-treated control group). Huperzine A significantly rescued the decrease of ATP level (p < 0.05 vs oligomeric Aβ_42_-treated group). There was no significant difference between control group and huperzine A treated alone group (p > 0.05).

ROS trigger the oxidative stress in cells and reflect the functional status of mitochondria. We detected the accumulated ROS with the probe H_2_DCF-diacetate to find out whether huperzine A could decrease the ROS level. As compared with control group, oligomeric Aβ_42_-exposed neurons showed higher DCF fluorescence intensity (Fig 3B, p < 0.05). Huperzine A treatment significantly reduced DCF fluorescence intensity (p < 0.05 vs oligomeric Aβ_42_-treated group). Similar to the effects observed in ATP assay, huperzine A treatment alone did not change the ROS level of neurons (p > 0.05).

Mitochondrial membrane potential is a sensitive indicator of mitochondrial stress, and the maintenance of Δψ_m_ is very important to energy conversion and other signaling pathways. We assessed whether huperzine A was involved in the stability of Δψ_m_ using the JC-1 dye. As shown in Fig 3C, the Δψ_m_ of oligomeric Aβ_42_ -treated group dropped over 20% as compared with the control group (p < 0.001 vs vehicle treated control group). Huperzine A mildly but statistical significantly (about 10%) ameliorated the abnormal decrease of Δψ_m_ (Fig 3C, p < 0.05 vs oligomeric Aβ_42_-treated group).

### Huperzine A decreased mitochondrial accumulation of Aβ_42_

**Fig 4:**
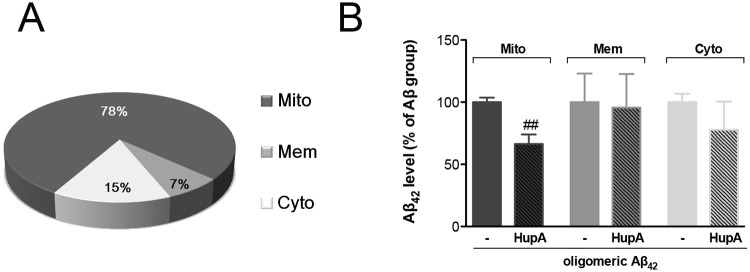
Huperzine A decreased mitochondrial accumulation of Aβ_42_. A. The schematic diagram of the proportion of mitochondria-enriched fraction Aβ_42_ in total intracellular Aβ_42_. B. Effect of huperzine A on the level of Aβ_42_ in different subcellular fractions (N = 4). Data were normalized by Aβ_42_-treated group and shown as mean ± SEM. ^##^p < 0.01 vs Aβ_42_ group. Huperzine A, mitochondria-enriched fraction, membrane fraction, cytosol fraction were respectively abbreviated as HupA, Mito, Mem, Cyto.

We separated mitochondria-enriched fraction, membrane fraction and cytosol fraction according to reported biochemical methods [32] and measured the Aβ level of different fractions through ELISA assay. We found that the majority of intracellular Aβ (about 78%) was distributed in the mitochondria-enriched fraction (Fig 4A). Huperzine A treatment significantly decreased the level of Aβ distributed in mitochondria-enriched fraction (p < 0.001 vs oligomeric Aβ_42_-treated group). Meanwhile, the Aβ level of membrane and cytosol fractions was not significantly influenced by huperzine A treatment (Fig 4B).

**Fig 5:**
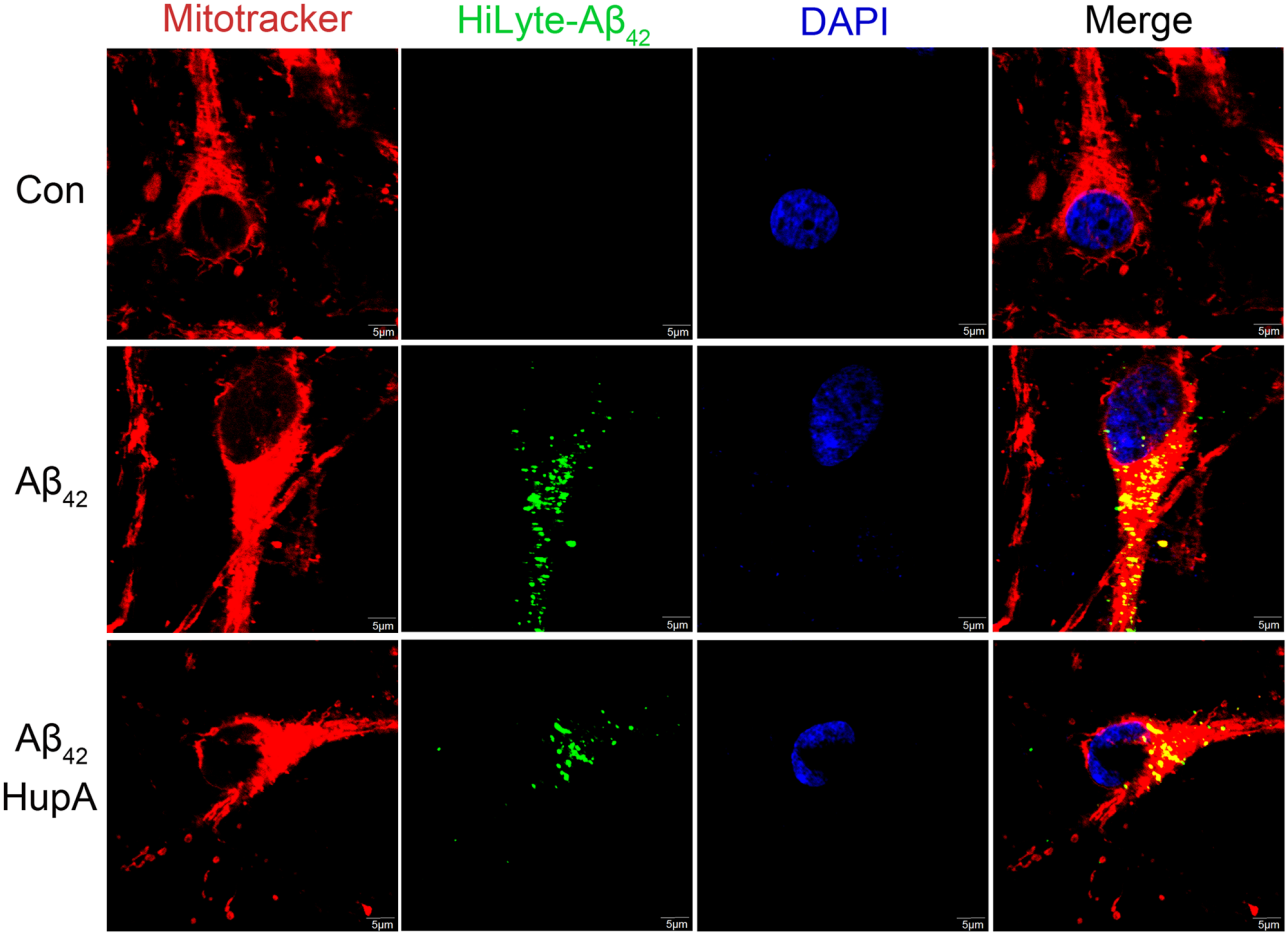
Huperzine A decreased HiLyte-Aβ_42_ fluorescent signals colocalized with mitochondrial marker in primary rat neurons. The representative confocal microscopy figures of each group. Scale bar: 5 μm.

To further confirm the results of mitochondrial Aβ, we applied mitotracker probe to label mitochondria of primary neurons. As shown in Fig 5, HiLyte-Aβ_42_ (green fluorescence) colocalized well with mitotracker (red fluorescence), and huperzine A treatment decreased fluorescent signals of HiLyte-Aβ_42_ and mitochondria colocalization, which was similar as the result obtained by ELISA.

## Discussion

More and more attentions have been attracted on addressing the contribution of intracellular Aβ in Aβ oligomers-induced neurotoxicity [7, 39], yielding vigorously growing researches on discovering associated novel therapeutic strategies for AD based on “Aβ cascade hypothesis”, during which employing small active molecules with potent pharmacological efficacy as probe is usually considered as an effective way [40]. As an extension of previous studies, we demonstrate for the first time that huperzine A, a natural product, possesses potent alleviative effect on oligomeric Aβ_42_-induced neuronal damage, which is closely associated with the reduced level of intracellular Aβ especially mitochondrial Aβ, as well as the ameliorated mitochondrial function.

A number of investigators now propose that Aβ oligomers, rather than Aβ fibers, may be mainly responsible for neuronal synaptic dysfunction both *in vivo* and *in vitro*, which ultimately induce neuronal network disruption [41]. On the other hand, considerable studies have demonstrated that Aβ_42_ is easier to form aggregates than Aβ_40_ [42, 43] and is the predominant component of senile plaques [44]. We therefore evaluate the effects of huperzine A on primary neuronal damage induced by oligomeric Aβ_42_. Consistent with previous findings that 1 μM Aβ oligomers notably induced the damage of primary cultured neurons [45, 46], we also observed that oligomeric Aβ_42_ significantly reduced MTT absorbance (Fig 1A). Post-administration of huperzine A significantly reversed the decreased MTT absorbance of primary cortical neurons induced by oligomeric Aβ_42_ exposure (Fig 1A), which was similar with our previous results in various cell lines and primary cortical neurons treated with the active fragment Aβ [15, 16]. No significant difference of SRB absorbance was found between each group (Fig 1B). The discrepancy between the results of MTT and SRB assays might be due to the different rationales behind the two methods: MTT assay measures the activity of succinate dehydrogenase—to reflect the metabolic activity of cells [47, 48], while SRB assay is based on the quantitative staining of cellular proteins—to reflect the cell number or density [49]. Therefore, the results of MTT and SRB assays demonstrated that oligomeric Aβ_42_ exposure significantly reduced the cell viability without causing obviously loss of primary neurons under our experimental conditions and the protective effect of huperzine A on the cell viability was not attributed to the cellular proliferation. Above results further suggested that the toxic effect of oligomeric Aβ_42_ and the protective effect of huperzine A may be very likely associated with mitochondrial function, as succinate dehydrogenase, detected in MTT assay, is a key enzyme of TCA cycle. It is worth mentioning that it was the first time we administrated huperzine A after the insult of exogenous Aβ, and the results will enrich our knowledge of the beneficial profiles of huperzine A in preventing or remedying Aβ-associated neurodegeneration.

A growing body of evidence indicates that intracellular Aβ is more toxic than extracellular Aβ [50, 51] and the level of intraneuronal Aβ is positively correlated with neuritic damage and synaptic alternations [52]. Therefore, intracellular Aβ could be one of the novel therapeutic targets for the treatment of AD, however, only a few interventions have been identified to specifically reduce the accumulation of Aβ intraneuronally and ameliorate the intracellular Aβ neurotoxicity [53]. One of the obstacles for efficient discovery of active compounds is the paucity of studies showing a convincing link between intracellular Aβ and neuronal death, we therefore systematically evaluated the changes of intraneuronal accumulation of Aβ in primary cortical neurons applying synthetic and oligomeric Aβ_42_. In our work, oligomeric Aβ accumulated into neurons time- and dose-dependently and correlated well with cell viability (Figure C in S1 File), which was consistent with earlier reports [32, 54]. Interestingly, by employing aforementioned cellular model, we demonstrated the co-occurrence of beneficial effects of huperzine A on neuronal survival and reduction of intracellular Aβ level. Our data suggest that the potent effect of huperzine A on reducing intracellular Aβ accumulation may effectively contribute to the protective effects of huperzine A against oligomeric Aβ_42_-induced neurotoxicity.

Mitochondria are believed as the major subcellular organelle for intracellular Aβ accumulation [21, 32], although Aβ also distributes in other sub-cellular compartments [55–57]. Similar as previous study [32], our results confirmed that mitochondria possessed the major part of intracellular Aβ (Fig 4A). Moreover, it is also reported that exogenous Aβ can be internalized into cells and colocalize with mitochondrial markers, which strongly influence mitochondrial respiratory function, ROS production rate, and mitochondria membrane potential [58, 59]. In our study, we firstly verified that the significant effects of huperzine A treatment on Aβ_42_ induced mitochondrial dysfunction, as determined by ATP, ROS and mitochondrial membrane potential measurement, happened concurrently with the decreased level of mitochondrial Aβ_42_ (Figs 4 and 5). Above results suggest that the beneficial role of huperzine A against oligomeric Aβ_42_-induced neuronal injury may be closely associated with its protection on mitochondria, which may be attributed to the reduced level of mitochondrial Aβ.

Considerable efforts have been put on impeding intracellular Aβ neurotoxicity through reducing the accumulation of intracellular Aβ. It is reported that the amount of intracellular Aβ could be influenced by various factors involved in Aβ-import process and Aβ-degrading process [7, 21], while blocking the entrance of extracellular Aβ into cytosol [53] and accelerating the clearance of intracellular Aβ [60] are considered as the two major intervention strategies. Our preliminary study found that the Aβ level in media of huperzine A-treated neurons showed increased tendency compared with that of Aβ-treated alone neurons. In our experiment, Aβ level in media was twenty-time higher than that in cell lysate, which should be sufficient for cellular uptake (data not shown). What’s more, huperzine A hardly influenced Aβ-degrading pathway in APP/PS1 transgenic mice according to our previous work [61]. Taken together, the reduction of intracellular Aβ level after huperzine A treatment was unlikely attributed to the degradation of Aβ. The precise mechanisms underlying the beneficial effects of huperzine A on reducing intracellular Aβ accumulation and neurotoxicity remain to be explored in future study. Nevertheless, our results provide important clues for an alternative way to hold back the progressing of intracellular Aβ neurotoxicity through decreasing the accumulation of intracellular or mitochondrial Aβ. What’s more, our results shed more light on the pharmacological effects of huperzine A, which may be used as a pharmacological probe either for developing anti-AD drugs or clarifying potential therapeutic targets against Aβ neurotoxicity.

## Supporting Information

S1 File Supporting figures. Aggregation state of Aβ_42_ (**Figure A**). Purity determination of subcellular fractions (**Figure B**). Intracellular Aβ_42_ accumulation and correlation analysis with cell viability (**Figure C**). The nonspecific band detected by 6E10 antibody (**Figure D**).
(DOCX)
