# Supplementary material for: Involvement of Intracellular and Mitochondrial Aβ in the Ameliorative Effects of Huperzine A against Oligomeric Aβ42-Induced Injury in Primary Rat Neurons
Source: PLoS One. 2015 May 29;10(5):e0128366. doi: 10.1371/journal.pone.0128366 (PMC4448999; doi:10.1371/journal.pone.0128366)
Supplement: S1 File — Aggregation state of Aβ42 (Figure A). Purity determination of subcellular fractions (Figure B). Intracellular Aβ42 accumulation and correlation analysis with cell viability (Figure C). The nonspecific band detected by 6E10 antibody (Figure D). (DOCX) [file pone.0128366.s001.docx]

**SUPPORTING INFORMATION**

**Involvement of intracellular and mitochondrial Aβ in the ameliorative effects of huperzine A against oligomeric Aβ_42_-induced injury in primary rat neurons**

Yun Lei^1^, Ling Yang^1^, Chun Yan Ye^1^, Ming Yan Qin^2^, Huai Yu Yang^2^, Hua Liang Jiang^2^,

Xi Can Tang^1^, Hai Yan Zhang^1,*^

^1^CAS Key Laboratory of Receptor Research, Shanghai Institute of Materia Medica, Chinese Academy of Science, Shanghai, China

^2^Drug Discovery and Design Center, State Key Laboratory of Drug Research, Shanghai Institute of Materia Medica, Chinese Academy of Sciences, Shanghai, China

*Correspondence to: Hai Yan Zhang, PhD, Shanghai Institute of Materia Medica, 555 Zu Chong Zhi Road, Zhangjiang Hi-Tech Park, Shanghai 201203, People’s Republic of China. Tel/Fax: +86-21-50806710; Email: [hzhang@simm.ac.cn](mailto:hzhang@simm.ac.cn).

The supplementary figures Figure A-D are as follows:


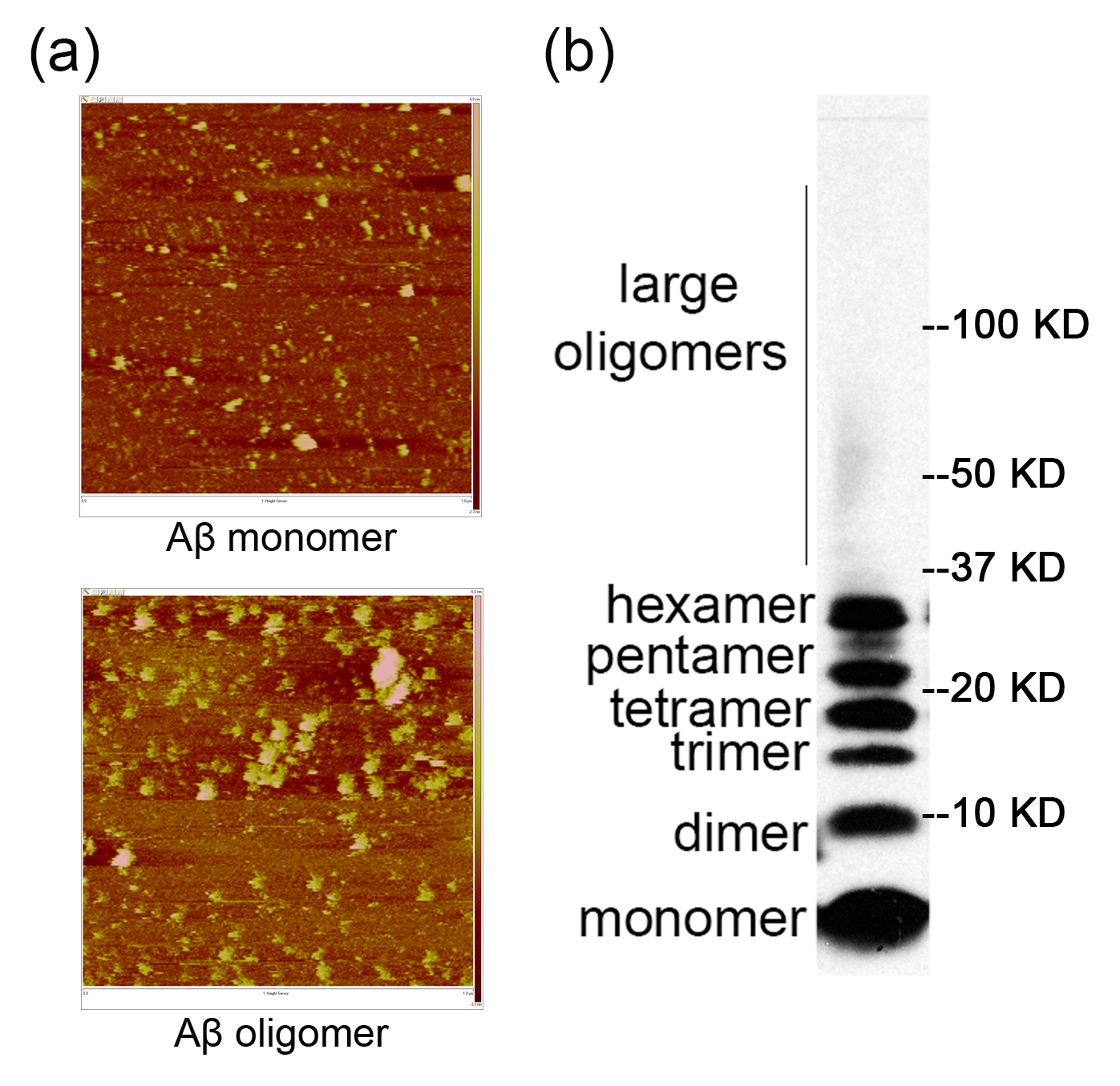


**Figure A. Aggregation state of Aβ_42_. (a).** Results from AFM. A BioScope Catalyst AFM (Bruke, USA) was used for AFM imaging. All Aβ samples of 1 mL were prepared on freshly cleaved mica (SPI Supplies, USA). The AFM imaging was conducted directly in solutions with the “scanasyst in fluid” mode. “Scanasyst in fluid+” probes (Veeco, USA) with spring constants of 0.7 N/M and resonance frequencies of 120-170 kHz were used. Images were captured at 500 × 500 nm size, a scan rate of 1 Hz, a target setpoint of 0.1 to 0.001 V, and a resolution of 256 × 256 pixels. **(b).** Results from western blotting analysis.


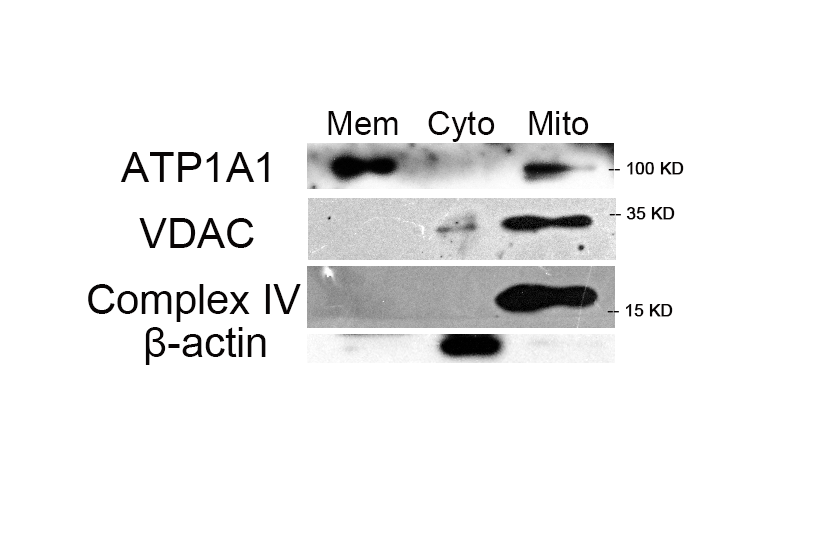


**Figure B. Purity determination of subcellular fractions.** Subcellular fractions were analyzed by western blotting. Na^+^/K^+^-ATPase catalytic component ATP1A1 was used as the marker of membrane fraction, and complex IV and VDAC were used as marker of mitochondria-enriched fraction. ATP1A1 (Proteintech, 14418-1-AP, mouse polyclonal antibody, 1:500), complex IV (Abcam, ab14744, mouse monoclonal antibody, 1:2000 dilution), VDAC (Calbiochem, PC548, rabbit polyclonal antibody, 1:5000 dilution).


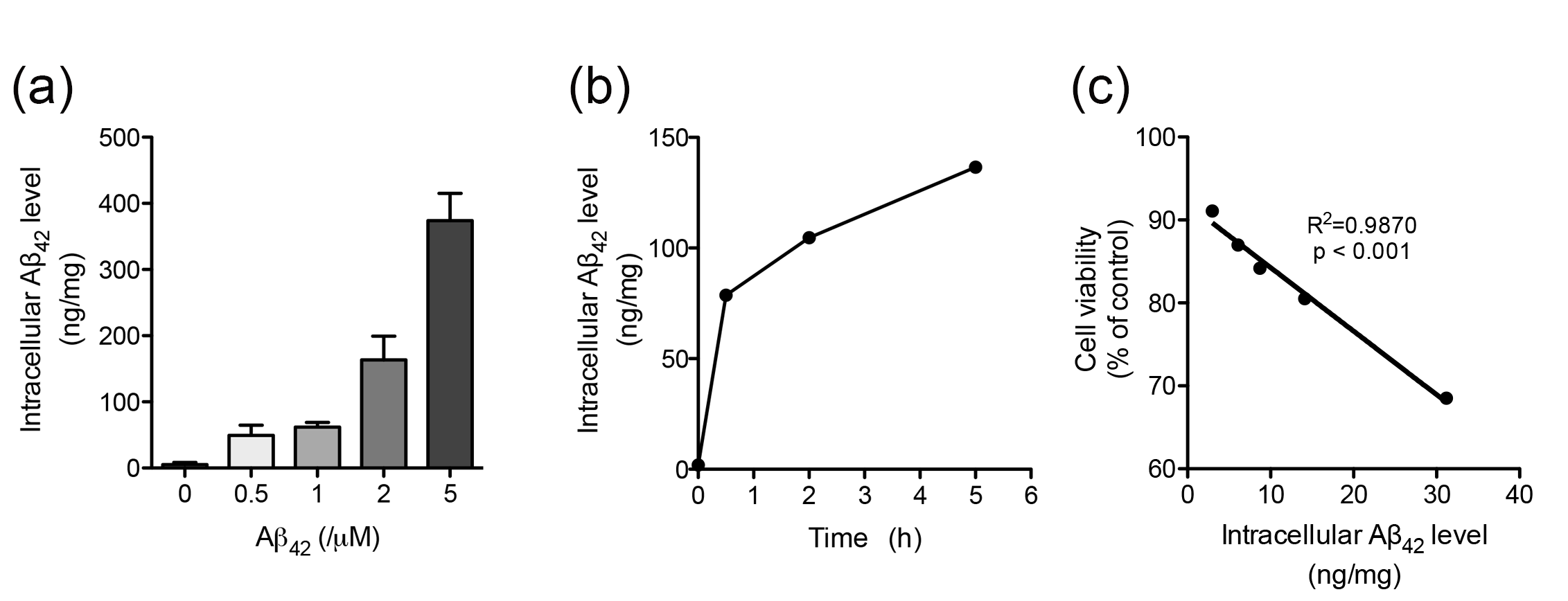


**Figure C. Intracellular Aβ_42_ accumulation and correlation analysis with cell viability. (a).** The level of Aβ_42_ accumulated in neurons exposed to different concentrations of oligomeric Aβ_42_ for 24 hours. **(b).** The level of Aβ_42_ accumulated in neurons exposed to 1.0 μM oligomeric Aβ_42_ for different time. **(c).** Correlation analysis between intracellular Aβ_42_ level and cell viability.

**
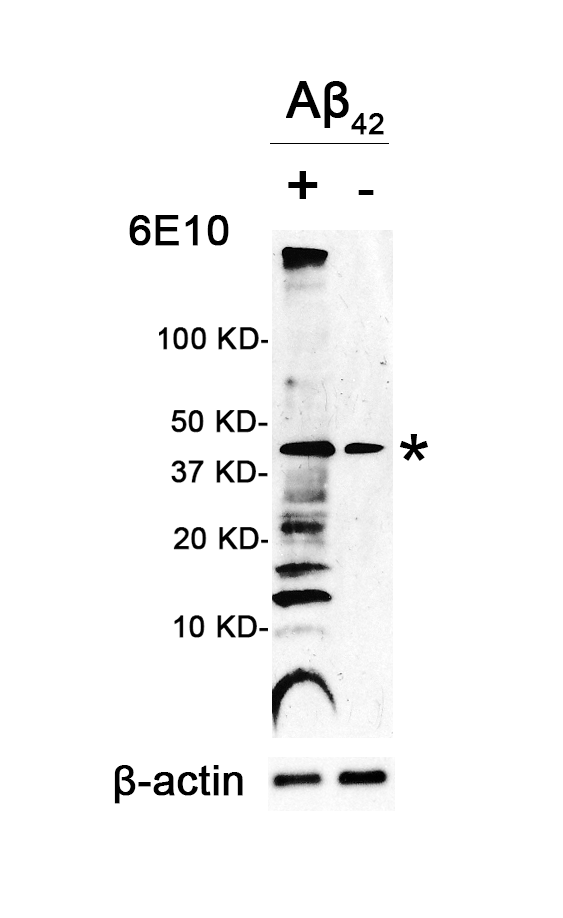
**

**Figure D. The nonspecific band detected by 6E10 antibody.** Asterisk denoted the non-specific band detected by the 6E10 antibody.
